# Wheat Bran Stir-Frying Reshapes the Metabolome of *Euryale Ferox* Seeds and Predicts Enhanced Bioactive Potential for Spleen and Kidney Tonifying

**DOI:** 10.3390/metabo16090669

**Published:** 2026-09-10

**Authors:** Panpan Li, Yaojia Zou, Yinghao Mao, Fan Ye, Tao Luo

**Affiliations:** College of Life Science, Jiangxi Science & Technology Normal University, Nanchang 330013, China; 2024020236@jxstnu.edu.cn (P.L.); z2940842437@icloud.com (Y.Z.); maoyinghao80@gmail.com (Y.M.); 1020170201@jxstnu.edu.cn (F.Y.)

**Keywords:** *Euryale ferox*, processing, metabolites, network pharmacology, molecular docking, Urolithin B, spleen and kidney tonifying

## Abstract

**Highlights:**

**What are the main findings?**

- Wheat bran stir-frying fundamentally reshapes the metabolome of *Euryale ferox* seeds, leading to a significant upregulation of 22 key bioactive metabolites.
- Urolithin B, a metabolite rarely found in plants, exhibits the most dramatic increase (168-fold), suggesting a possible processing-induced chemical transformation that warrants further investigation.

**What are the implications of the main findings?**

- The upregulated metabolites, particularly Urolithin B, Tangeretin, and Syringetin, were predicted to strongly bind to core targets (e.g., MMP2, MMP9), suggesting a potential mechanism for the enhanced anti-tumor and anti-diabetic activities.
- This study provides a computational basis for understanding the traditional processing practice, linking traditional knowledge with modern scientific evidence and highlighting the potential of Urolithin B as a critical quality marker.

**Abstract:**

Background: *Euryale ferox* Salisb. seed (Euryales Semen) is a traditional Chinese medicine whose therapeutic application is modulated by wheat bran stir-frying, a process that enhances its effects on tonifying the spleen and kidney. The molecular mechanisms and alterations in bioactive components underlying this processing-enhanced efficacy remain to be fully elucidated. This study aimed to systematically investigate the impact of wheat bran stir-frying on the metabolite profile of Euryales Semen and to reveal its potential pharmacological mechanisms. Methods: An integrated strategy combining UPLC-MS/MS-based metabolomics, network pharmacology, and molecular docking was employed. First, differential metabolites between raw and processed Euryales Semen were identified and quantified. Second, a “metabolite-target-disease” network was constructed to predict core targets and pathways. The docking protocol was validated by re-docking of co-crystallized ligands, with all RMSD values below 2.0 Å. Finally, molecular docking was used to predict the binding interactions between key metabolites and potential protein targets. Results: A total of 1597 metabolites were identified, and 244 were defined as differentially expressed (VIP > 1, |log2(FC)| ≥ 1). Processing significantly upregulated 19 of the 22 key metabolites identified, with Urolithin B showing the most dramatic increase (168-fold), which was identified with Level 1 confidence using an authentic standard. Network pharmacology analysis identified 10 core targets (e.g., MMP2, MMP9, AKR1B1) primarily associated with tumors, diabetes, and other chronic diseases. Molecular docking predicted strong binding affinities (<−9.0 kcal·mol^−1^) between these core targets and key upregulated metabolites, including Urolithin B, Tangeretin, and Syringetin. Conclusions: This study suggests that wheat bran stir-frying fundamentally reshapes the metabolic profile of Euryales Semen, leading to the enrichment of bioactive metabolites (e.g., Urolithin B) and suggesting a potential for enhanced tonifying, anti-tumor, and anti-diabetic activities that warrants further experimental investigation. The dramatic increase in the content of Urolithin B, a metabolite not commonly found in plants, raises the hypothesis of a processing-induced chemical transformation, which warrants further experimental validation, and highlights its potential as a critical component for the herb’s efficacy. The findings offer a preliminary computational basis for understanding traditional processing practices, linking traditional knowledge with modern scientific evidence.

## 1. Introduction

The processing (Paozhi) of herbal materials is a cornerstone of Traditional Chinese Medicine (TCM), serving to enhance therapeutic efficacy, reduce potential toxicity, and tailor the properties of medicinal substances for specific clinical applications [1]. Despite its historical importance, the molecular mechanisms underpinning these processing-induced transformations are often not fully understood, representing a significant gap in the scientific validation of TCM practices. A prime example is the seed of *Euryale ferox* Salisb. (Euryales Semen), a highly valued herb and food item often referred to as the “ginseng of the water” [2]. According to the Chinese Pharmacopoeia, the clinical application of Euryales Semen is differentiated based on its processing state: the raw form is traditionally used to consolidate essence and reduce excessive vaginal discharge, whereas stir-frying with wheat bran modifies its property to strengthen the spleen and stomach, thereby improving its function in tonifying the spleen and kidney [3,4]. This empirically established shift in therapeutic indication strongly implies that profound alterations in its chemical composition occur during processing.

Modern scientific investigations have begun to unravel the complex composition of Euryales Semen, revealing a rich reservoir of nutrients and bioactive compounds, including proteins, vitamins, lignans, flavonoids, and cyclic dipeptides [5]. These constituents are believed to underpin its broad spectrum of reported pharmacological activities, such as antioxidant, anti-diabetic, anticancer, and anti-atherosclerosis effects [6]. Critically, secondary metabolites are increasingly recognized as the fundamental material basis for these physiological activities [7]. While previous research has provided valuable insights, a systematic and comprehensive analysis of how wheat bran stir-frying—a critical processing step—reshapes the global metabolic profile of Euryales Semen and consequently modulates its pharmacological network is still lacking. The fundamental question of which specific metabolites are altered, how these changes correlate with the enhanced tonifying effects, and through what molecular targets they operate remains largely unanswered.

To address these questions, modern omics and bioinformatics approaches offer powerful tools. Metabolomics provides a high-throughput and sensitive platform for the systematic identification and quantification of metabolites [8], making it ideal for capturing the chemical consequences of processing. Network pharmacology, with its holistic perspective, aligns perfectly with the multi-component, multi-target nature of TCM, enabling the construction of interactive networks between drug components and biological targets [9]. Furthermore, molecular docking serves as a valuable computational technique for simulating and validating the binding interactions between key bioactive compounds and their potential protein targets [10]. The integration of these three methodologies has proven successful in elucidating the processing mechanisms of other TCM materials, such as *Pinellia ternate* [11] and *Gardenia jasminoides* [12], demonstrating its efficacy in linking compositional changes to therapeutic outcomes.

**Figure 1:**
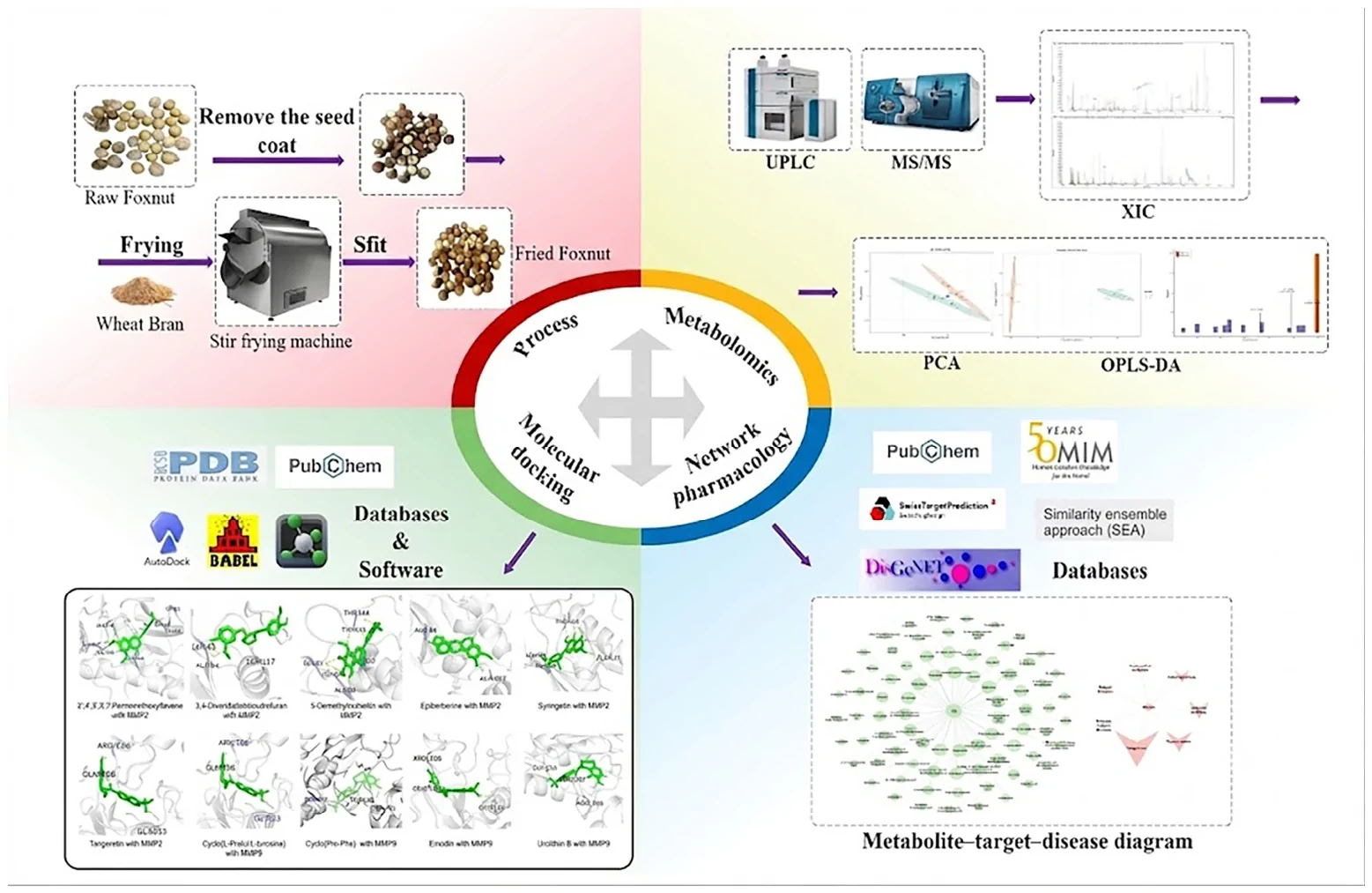
Experimental flow chart. Experimental flow chart. Pink boxes indicate the processing steps of Euryale ferox seeds; yellow boxes represent the integrated analytical methods (UPLC-MS/MS-based metabolomics); blue boxes denote database mining and enrichment of core diseases and targets; green boxes show molecular docking and visualization.

Therefore, this study was designed to systematically investigate the impact of wheat bran stir-frying on the metabolite composition and pharmacological potential of Euryales Semen by employing an integrated strategy of UPLC-MS/MS-based metabolomics, network pharmacology, and molecular docking. We aimed to: (1) comprehensively identify and quantify the differential metabolites between raw and processed Euryales Semen; (2) screen for drug-like bioactive metabolites and construct a “metabolite-target-disease” network to predict the core targets and pathways; and (3) predict the interactions between key metabolites and potential targets through molecular docking. Our findings point to a set of critical upregulated metabolites, most notably Urolithin B, and delineate their network of actions, thereby providing a potential mechanistic basis for the processing-enhanced efficacy of Euryales Semen and bridging traditional processing knowledge with contemporary scientific evidence (Figure 1).

## 2. Materials and Methods

### 2.1. Plant Materials and Chemicals

Mature seeds of *Euryale ferox* Salisb. (Euryales Semen) were harvested from the Santang Planting Base (Yugan County, Shangrao City, Jiangxi Province, China). The plant material was authenticated by Professor Xide Ye of Jiangxi University of Chinese Medicine.

HPLC-grade methanol and acetonitrile were purchased from Merck (Darmstadt, Germany). HPLC-grade formic acid was obtained from Shanghai Aladdin Biochemical Technology Co., Ltd. (Shanghai, China). Ultrapure water was prepared using a Milli-Q water purification system (Millipore, Bedford, MA, USA).

### 2.2. Processing via Wheat Bran Stir-Frying

The processing of Euryales Semen followed the wheat bran stir-frying procedure as described in the Chinese Pharmacopoeia (2020 edition, General Chapter 0213), with the specific conditions (200 °C, 2.5 min, 1.3 g wheat bran per 10 g seed) optimized via response surface methodology in our previous study [13]. In brief, 10.0 g of raw Euryales Semen was stir-fried with 1.3 g of wheat bran in a preheated copper pot at 200 °C for 2.5 min.

### 2.3. Widely Targeted Metabolomics Analysis Using UPLC-MS/MS

#### 2.3.1. Sample Extraction

Both raw and processed samples were lyophilized using a freeze dryer(Scientz-100F, Scientz, Ningbo, China) and then ground into a homogeneous powder using a grinding mill(MM400, Retsch, Haan, Germany) at 30 Hz for 1.5 min. Subsequently, 50 mg of the powdered material was accurately weighed using an electronic balance (MS105DM, Mettler Toledo, Zurich, Switzerland) and extracted with 1200 μL of a pre-cooled (−20 °C) extraction solution (70% methanol in water, *v*/*v*) containing an internal standard mixture (Micotrol, MetWare, Wuhan, China). The mixture was vortexed every 30 min for 30 s, repeated six times. After centrifugation at 12,000 rpm for 3 min, the supernatant was collected, filtered through a 0.22 μm microporous membrane, and transferred into an injection vial for UPLC-MS/MS analysis. A quality control (QC) sample was prepared by pooling equal volumes of all individual extracts to monitor instrument stability. Each group consisted of three independent biological replicates (n = 3), representing separate stir-frying batches. All metabolite contents were normalized to the dry weight of the lyophilized powder, thereby controlling for potential concentration effects due to moisture loss during the stir-frying process.

#### 2.3.2. UPLC-MS/MS Conditions

Chromatographic separation was performed on an Agilent SB-C18 column (1.8 μm, 2.1 × 100 mm) maintained at 40 °C. The mobile phase consisted of (A) ultrapure water with 0.1% formic acid and (B) acetonitrile with 0.1% formic acid. The flow rate was set at 0.35 mL/min with an injection volume of 2 μL. The elution gradient parameters are detailed in Table 1.

Mass spectrometric detection was conducted on a QTRAP^®^ 6500+ mass spectrometer (SCIEX, Framingham, MA, USA) equipped with an electrospray ionization (ESI) source. The source temperature was 500 °C. The ion spray voltage was set at 5500 V (positive mode) and −4500 V (negative mode). Ion source gas I (GSI), gas II (GSII), and curtain gas (CUR) were set at 50, 60, and 25 psi, respectively. Data acquisition was performed in multiple reaction monitoring (MRM) mode. The declustering potential (DP) and collision energy (CE) for each MRM transition were optimized individually.

#### 2.3.3. Data Processing and Metabolite Identification

The raw data files from UPLC-MS/MS were processed using Analyst 1.6.3 software (SCIEX, USA) for peak integration and correction. Metabolite identification was performed by comparing the secondary mass spectrometric data with the self-built MWDB database (MetWare Biotechnology Co., Ltd., Wuhan, China), which contains over 1600 metabolites. The identification confidence was classified into three levels according to the following criteria: Level 1, MS/MS spectrum and retention time matched the database with a score ≥ 0.7; Level 2, MS/MS spectrum and retention time matched with a score between 0.5 and 0.7; Level 3, Q1, Q3, retention time, declustering potential (DP), and collision energy (CE) matched the database entries. The identification of Urolithin B was confirmed at Level 1 using an authentic standard for retention time and MS/MS spectral matching. Isotopic signals and redundant ions (including K^+^, Na^+^, and NH_4_^+^ adducts) were excluded from the analysis. Quality control (QC) samples were prepared by pooling equal volumes of all individual extracts and injected every 10 samples throughout the analytical run to monitor instrument stability; metabolites with a relative standard deviation (RSD) > 30% in QC samples were excluded from subsequent analysis. In total, 47 metabolites were removed using this filtering criterion. The overall QC RSD distribution showed that more than 85% of the detected metabolites had RSD values below 0.5, and more than 75% had RSD values below 0.3, indicating good analytical reproducibility. All data were log2-transformed and mean-centered prior to multivariate statistical analysis. The UPLC-MS/MS data acquisition and primary data processing, including peak integration, metabolite identification, and quantification, were performed by MetWare Biotechnology Co., Ltd. (Wuhan, China) as a fee-for-service. All subsequent statistical analyses (PCA, OPLS-DA, differential metabolite screening) were performed independently by the authors.

#### 2.3.4. Statistical Analysis

The processed dataset was log2-transformed and mean-centered prior to multivariate statistical analysis. Unsupervised principal component analysis (PCA) was performed using the prcomp function in R (www.r-project.org). Supervised orthogonal partial least squares–discriminant analysis (OPLS-DA) was conducted using the ropls package (version 1.6.2) in R. The model was validated with a 200-permutation test to prevent overfitting. Differential metabolites between the sr and sr-p groups were screened based on a variable importance in projection (VIP) value > 1 from the OPLS-DA model and an absolute log2 fold change (|log2(FC)|) ≥ 1. The OPLS-DA model yielded R^2^Y = 0.999 and Q^2^ = 0.781, with permutation test intercepts of R^2^ = 0.123 and Q^2^ = −0.289.

### 2.4. Network Pharmacology Analysis Methods

#### 2.4.1. Screening of Bioactive Metabolites

The Canonical SMILES of the 244 differential metabolites were retrieved from the PubChem database (https://pubchem.ncbi.nlm.nih.gov). Drug-likeness assessment was performed using the SwissADME web tool (http://www.swissadme.ch/), adhering to Lipinski’s Rule of Five criteria: molecular weight between 180 and 500, hydrogen bond donors ≤ 5, hydrogen bond acceptors ≤ 10, consensus Log P ≤ 5, and rotatable bonds ≤ 10 [14]. This screening yielded 80 metabolites with potential pharmacological activity.

#### 2.4.2. Prediction of Compound Targets and Disease Targets

The 2D structures of the drug-like metabolites were submitted to the SwissTargetPrediction (http://www.swisstargetprediction.ch/) [15,16] and SEA (https://sea.bkslab.org/) [17] databases to predict potential protein targets. Only targets for Homo sapiens with a probability > 0 (SwissTargetPrediction) or a *p*-value < 0.05 (SEA) were retained. Although a relatively permissive threshold (probability > 0) was used for initial target collection to ensure broad coverage of potential pharmacological targets, this approach has been adopted in published network pharmacology studies, including a study on Tribuloside in acute lung injury [18]. The subsequent network topology filtering (based on Degree, Betweenness, and Closeness) and molecular docking with stringent binding energy cutoffs and ligand efficiency analysis served as multi-step refinement processes, effectively compensating for the initial permissive cutoff. The retrieved targets were standardized using the UniProt database (https://www.uniprot.org/) [19].

Disease targets related to diabetes mellitus, hypertension, kidney disease, myocardial ischemia, neoplasms, atherosclerosis, and immune system diseases were collected from DisGeNET (https://www.disgenet.org) [20] and OMIM (https://www.omim.org) [21] databases. Targets with a score ≥ 0.1 in DisGeNET and those marked with an asterisk (*) in OMIM were selected.

#### 2.4.3. Network Construction and Analysis

A Venn diagram was generated to identify the common targets between the compound-related and disease-related targets. A “metabolite-target-disease” network was then visualized using Cytoscape software (version 3.9.0). Key metabolites and core targets were identified based on network topology parameters (Degree, Betweenness, Closeness), calculated using the built-in NetworkAnalyzer tool in Cytoscape.

### 2.5. Molecular Docking

The 3D structures of the key metabolites were downloaded in SDF format from PubChem and converted to mol2 format using Open Babel (version 3.1.1). The crystal structures of the target proteins were retrieved from the RCSB PDB database (http://www.rcsb.org/). For each target, the following criteria were applied for structure selection: (1) preference was given to structures derived from Homo sapiens; (2) preference was given to X-ray diffraction structures with a resolution better than 2.5 Å; (3) preference was given to holo structures containing representative ligands in the binding pocket; and (4) among comparable structures, the more recently deposited ones were prioritized. Based on these criteria, the following PDB structures were selected for docking analysis: AKR1B1 (PDB ID: 8FH8,), AKT1 (PDB ID: 8UW9) [22], ALOX5 (PDB ID: 6N2W) [23], GSK3B (PDB ID: 9X2Q) [24], IGF1R (PDB ID: 8PYN), MMP2 (PDB ID: 7XJO) [25], MMP9 (PDB ID: 8K5Y) [26], PARP1 (PDB ID: 7ONS) [27], PIK3CG (PDB ID: 6AUD) [28], and PTGS2 (PDB ID: 5F19) [29].

Prior to docking, all protein structures were prepared using PyMOL (version 2.6.0) and AutoDock Tools (version 1.5.7): water molecules and non-essential ligands were removed; polar hydrogen atoms were added; Gasteiger charges were assigned; and protonation states were adjusted to physiological pH. Crystallographic water molecules were removed to avoid potential steric hindrance in the binding pocket and to prevent interference with the accurate calculation of binding energies, as non-specifically bound water molecules may introduce false-positive results in docking predictions. The binding pockets were defined based on the co-crystallized ligand positions or literature-reported active site residues, and grid boxes were generated using AutoGrid to fully encompass the entire known binding pocket. Molecular docking was carried out using AutoDock4 [30] through the AutoDockTools-1.5.7 graphical interface, with the number of genetic algorithm runs (ga_run) set to 10. For each ligand–target pair, 10 docking poses were generated, and the conformation with the lowest binding energy was selected for subsequent analysis. A total of 22 key metabolites were docked against 10 target proteins. The docking results were visualized using PyMOL, and protein–ligand interactions, including hydrogen bonds and hydrophobic contacts, were analyzed.

To validate the reliability of our docking protocol, we performed re-docking of the co-crystallized ligands into their respective protein structures for all ten target proteins using the same parameters as those employed in the formal docking analysis. If all RMSD values were below 2.0 Å, this would confirm that our docking parameters and grid box definitions could reliably reproduce the native binding conformations.

The widely targeted metabolomic analysis, including sample preparation, UPLC-MS/MS analysis, data acquisition, and primary data processing (metabolite identification and quantification), was performed by MetWare Biotechnology Co., Ltd. (Wuhan, China) as a fee-for-service. The authors provided all plant materials, designed the study, and performed all subsequent statistical and bioinformatic analyses (including network pharmacology and molecular docking). All network pharmacology and molecular docking analyses described in this section were performed independently by the authors.

## 3. Results

### 3.1. Metabolomic Analysis of Euryales Semen Before and After Stir-Frying with Wheat Bran

**Figure 2:**
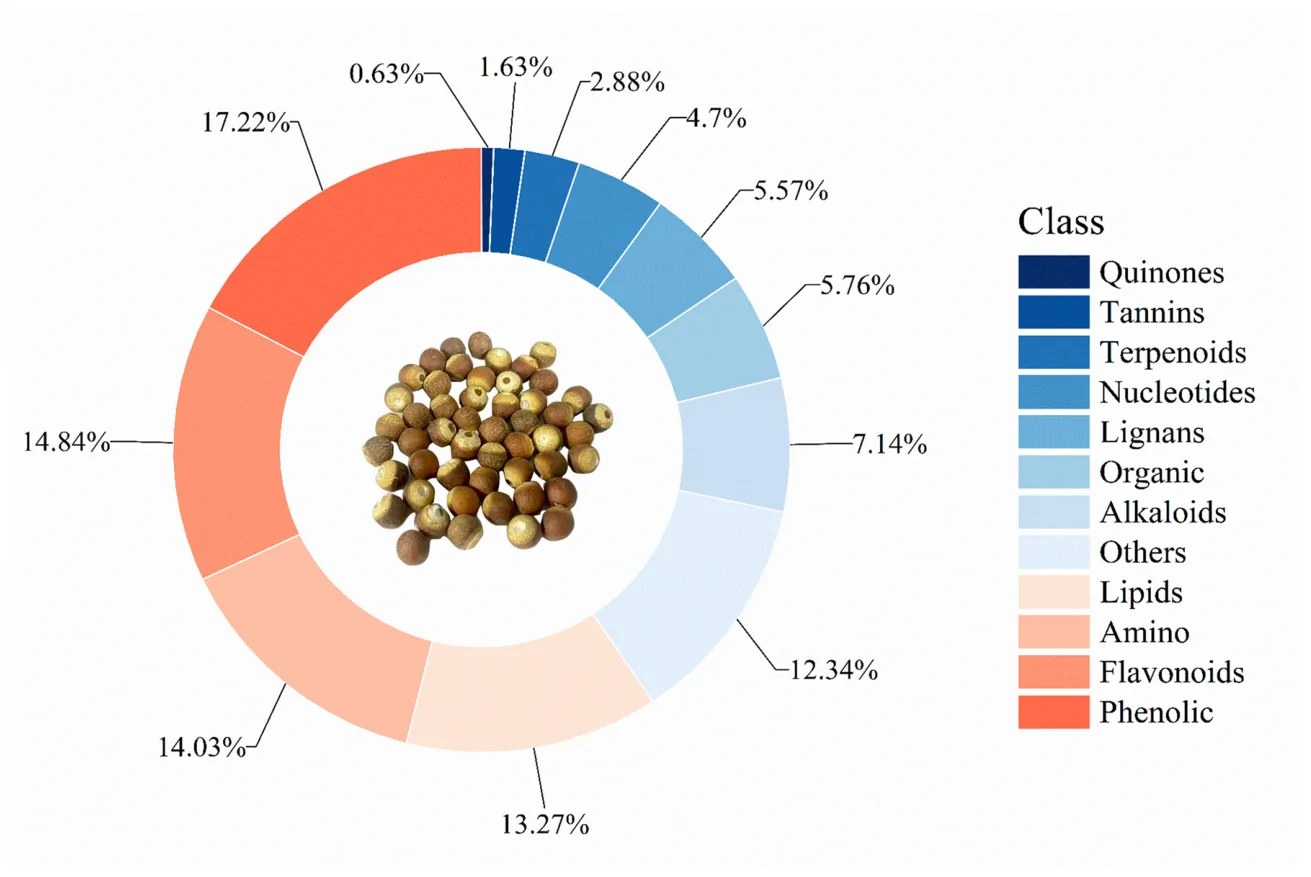
The metabolite classes form a circular graph.

The metabolic profiles of raw and processed Euryales Semen were comprehensively characterized using UPLC-MS/MS. A total of 1597 metabolites were identified and categorized into 12 classes, with phenolic acids (275), flavonoids (237), and amino acids and derivatives (224) being the most abundant (Figure 2).

#### 3.1.1. Metabolic Profiling and Group Separation

**Figure 3:**
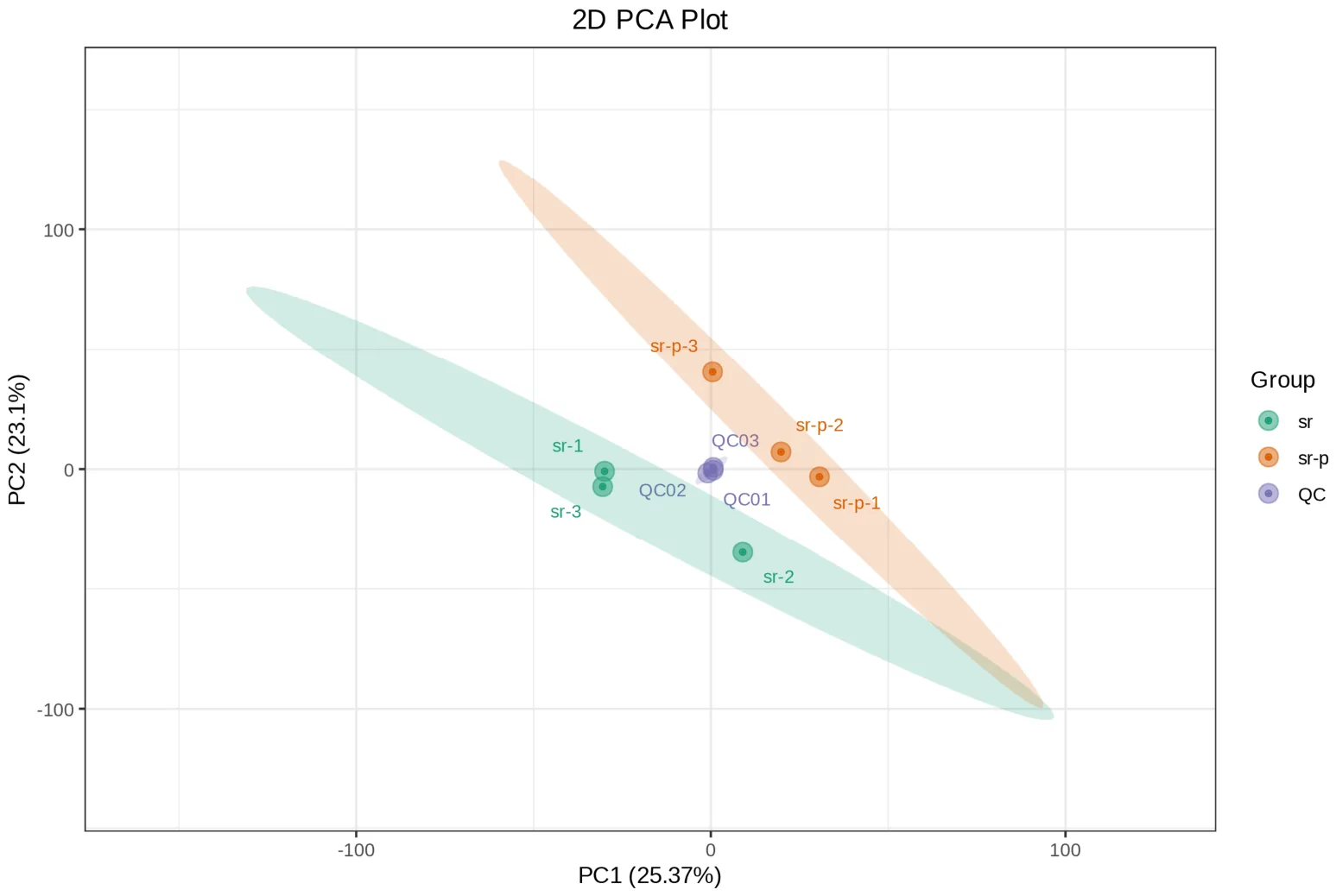
Score plots of PCA for Euryales Semen samples of sr, sr-p and quality control (QC) samples. PC1 and PC2 represent the first and second principal components, respectively.

**Figure 4:**
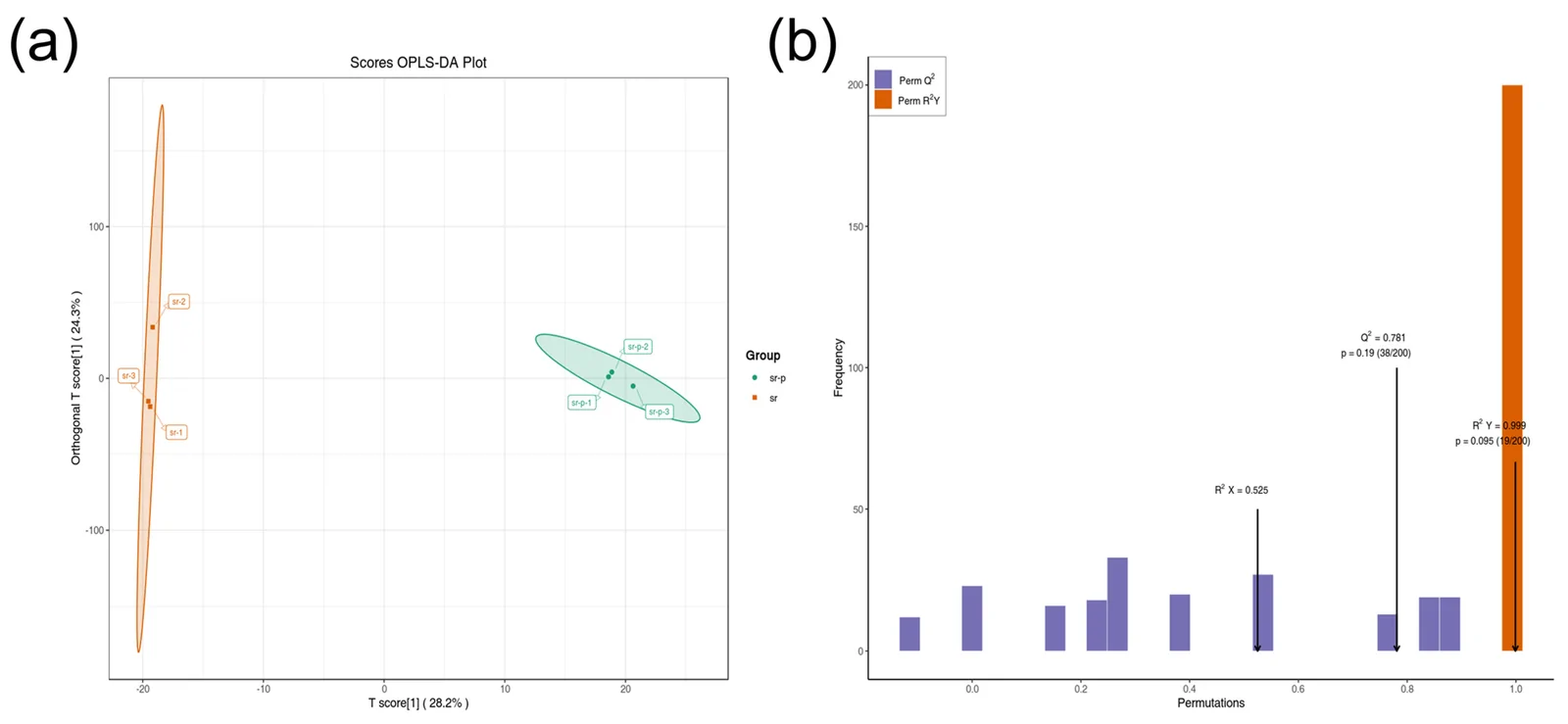
(**a**) is the score plots of the OPLS-DA model; the X-axis and Y-axis represent the predictive principal component and the orthogonal principal component, respectively. The plots in (**b**) are the model validation of (**a**).

Multivariate statistical analysis revealed significant metabolic differences between the raw (sr) and processed (sr-p) Euryales Semen. Principal component analysis (PCA) demonstrated tight intra-group clustering and a clear separation trend between the sr and sr-p groups (Figure 3), indicating excellent reproducibility and a substantial processing effect. The orthogonal partial least squares–discriminant analysis (OPLS-DA) model further confirmed this distinct separation (Figure 4a). The model’s validity was supported by high performance metrics (R^2^Y = 0.999, Q^2^ = 0.781) and a permutation test (200 iterations) showing no overfitting (Figure 4b). The permutation test intercepts were R^2^ = 0.123 and Q^2^ = −0.289. Given the limited sample size (n = 3 per group), leave-one-out cross-validation was not performed; however, the Q^2^ value substantially exceeds the commonly accepted threshold of 0.5, and the negative Q^2^ intercept from the permutation test collectively indicate that the model has good predictive ability and is not overfitted.

#### 3.1.2. Key Differential Metabolites Induced by Processing

Comparative analysis identified 244 differential metabolites based on the criteria of VIP > 1 and |log2(FC)| ≥ 1. As indicated in the technical report from our metabolomics service provider, the combination of multivariate (VIP) and univariate (FC) filtering represents a widely accepted approach in widely targeted metabolomics studies for effectively identifying metabolites with substantial abundance changes while reducing false positives. Although FDR-adjusted *p*-values were calculated for reference, they were not employed as a formal filtering criterion, as the primary objective of this study was to identify metabolites with pronounced abundance changes that could serve as potential markers for processing-induced bioactivity. The *p*-values for the 22 key metabolites have been compiled in Table S2 for reference. Their classification is summarized in Table 2. The processing led to a notable upregulation of metabolites in several key classes, including phenolic acids (40), flavonoids (40), organic acids (22), and lignans and coumarins (22). Among the significantly upregulated flavonoids were several polymethoxyflavones (PMFs), such as 3′,4′,5′,5,7-Pentamethoxyflavone and 5,6,7,8,3′,4′-Hexamethoxyflavone. The most strikingly upregulated compound was Urolithin B, which was identified with Level 1 confidence using an authentic standard, and its relative content increased dramatically, establishing it as a major metabolite in the processed samples.

### 3.2. Network Pharmacology Analysis

To elucidate the pharmacological implications of the metabolic changes, network pharmacology analysis was performed. From the 244 differential metabolites, 80 were screened as drug-like bioactive compounds and were associated with 947 potential protein targets. A search of disease databases yielded 3613 targets related to seven diseases (diabetes mellitus, hypertension, etc.), and the intersection of these target sets revealed 522 common targets (Figure S1).

**Figure 5:**
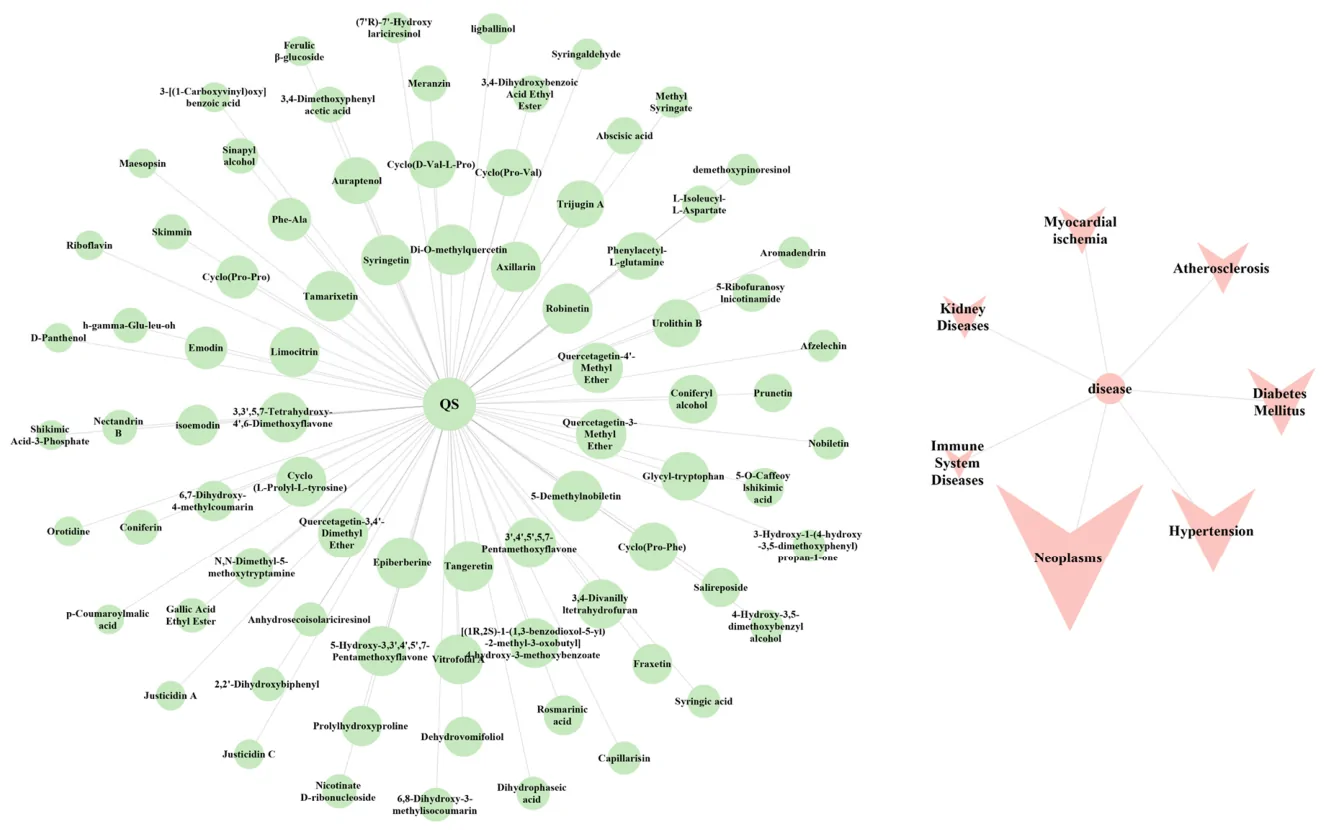
Metabolite–target–disease diagram. Green nodes represent metabolites, pink nodes are diseases, and the target has been hidden. Edges in the network are used to connect metabolites and targets, and diseases and targets. The size of nodes depends on their degree value; the larger the node is, the greater the degree value.

A “metabolite-target-disease” network was subsequently constructed, comprising 610 nodes and 3853 edges (Figure 5), illustrating the multi-component, multi-target nature of Euryales Semen’s activity. Using the average values of Degree, Betweenness, and Closeness as screening criteria, 22 key metabolites—including CHEMBL3978563, 3′,4′,5′,5,7-Pentamethoxyflavone, Cyclo (L-Pro-L-Tyr), Cyclo (Pro-Phe), Syringetin, Tangeretin, and Urolithin B—were identified. Concurrently, 10 core targets were pinpointed: AKR1B1, AKT1, ALOX5, GSK3B, IGF1R, MMP2, MMP9, PARP1, PIK3CG, and PTGS2. Notably, the node representing neoplasms (tumors) possessed the highest Degree value in the disease network (Degree = 391), implying a possible association with anti-tumor bioactivity post-processing, although this prediction requires further experimental validation. As a quality control measure, the analytical reproducibility of the metabolomics data was also assessed; QC samples exhibited good reproducibility, with over 85% of metabolites showing RSD < 0.5 and over 75% showing RSD < 0.3, and 47 metabolites with RSD > 30% in QC samples were excluded from subsequent analysis.

### 3.3. Molecular Docking Analysis

**Figure 6:**
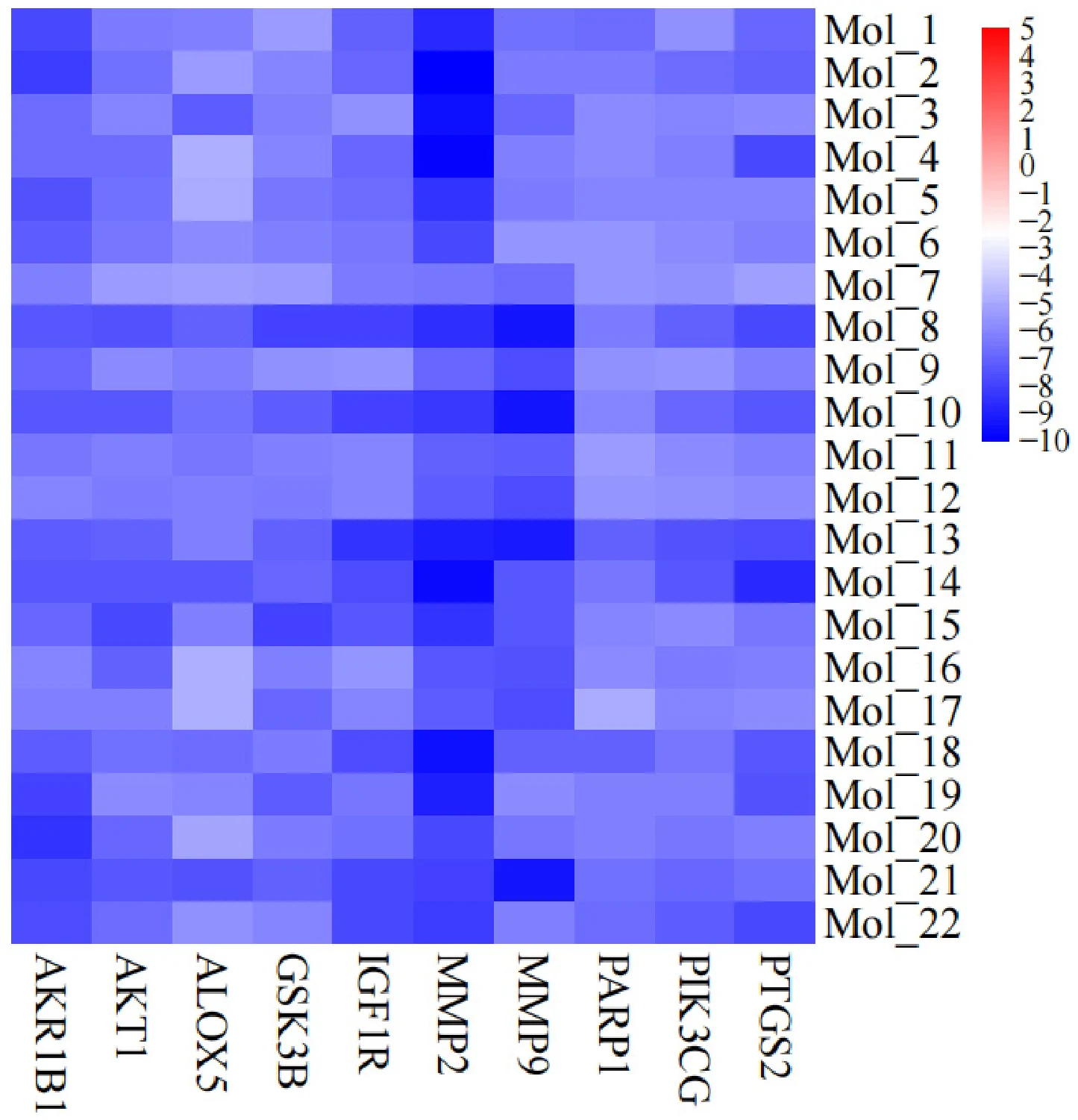
Heatmap of molecular docking affinity.

Prior to the formal docking analysis, re-docking validation was performed for all ten target proteins to confirm the reliability of our docking protocol. The RMSD values for AKR1B1 (PDB ID 8FH8), AKT1 (PDB ID 8UW9), ALOX5 (PDB ID 6N2W), GSK3B (PDB ID 9X2Q), IGF1R (PDB ID 8PYN), MMP2 (PDB ID 7XJO), MMP9 (PDB ID 8K5Y), PARP1 (PDB ID 7ONS), PIK3CG (PDB ID 6AUD), and PTGS2 (PDB ID 5F19) were 0.69, 1.87, 0.92, 1.64, 1.21, 0.62, 1.08, 0.74, 1.41, and 0.97 Å, respectively. All values were below 2.0 Å, indicating that our docking parameters and grid box definitions were capable of reliably reproducing the native binding poses. Molecular docking was employed to predict the interactions between the 22 key metabolites and the 10 core targets. It should be noted that docking provides a hypothesis-generating framework for potential interactions and does not confirm binding affinity or biological relevance. The obtained binding affinity data were organized into a Summary of molecular docking results for key bioactive metabolites identified from metabolomics analysis (Table 3) and visualized as a heatmap (Figure 6). Notably, 137 out of the 220 docking combinations (62.3%) exhibited strong binding affinities (binding energy < −6.0 kcal·mol^−1^). To further assess whether the observed binding energies reflect genuine target affinity rather than a size-related artifact, we calculated ligand efficiency (LE = binding energy / heavy atom count) [31,32] for the key metabolite–target pairs. The LE values for the top hits were: −0.37 for 3′,4′,5′,5,7-Pentamethoxyflavone–MMP2, −0.38 for 5-Demethylnobiletin–MMP2, −0.38 for Syringetin–MMP2, −0.59 for Urolithin B–MMP9, −0.34 for Tangeretin–MMP2, and −0.46 for Emodin–MMP9. All values were below −0.3 kcal·mol^−1^ per heavy atom, indicating favorable ligand efficiency and supporting that the predicted strong binding affinities are not merely a consequence of ligand size. The 22 key metabolites showed particularly strong predicted binding to three targets: MMP2, MMP9 and AKR1B1.

**Figure 7:**
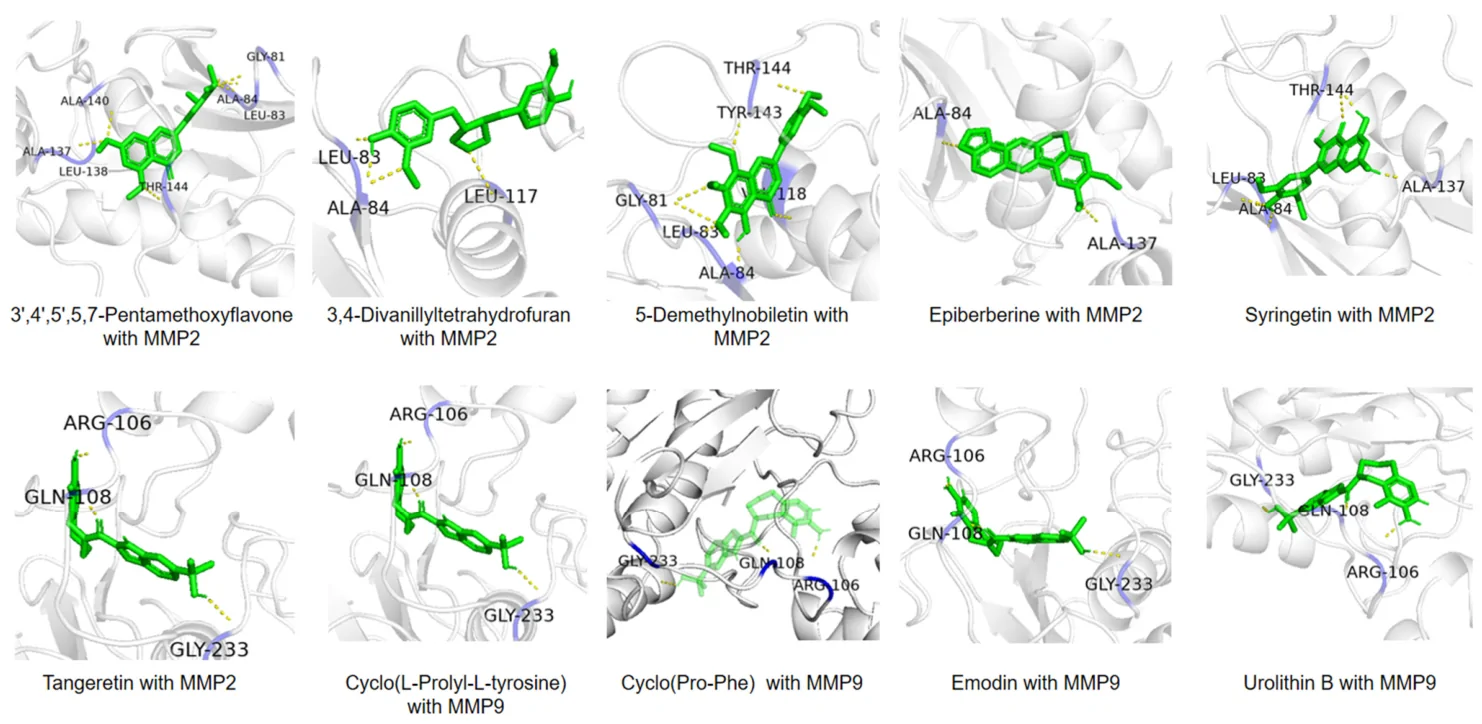
Molecular docking poses of key metabolites with core targets, representing the conformations with the highest binding affinity (<−9.0 kcal·mol^−1^). Color coding: green, ligand; blue, key residues; yellow dashed lines, hydrogen bonds; gray surface, molecular surface of the binding site.

For detailed visualization, complexes with binding energies less than −9.0 kcal·mol^−1^ were selected (Figure 7). The results suggested that ten key metabolites—3′,4′,5′,5,7-Pentamethoxyflavone, 3,4-Divanilyltetrahydrofuran, 5-Demethylnobiletin, Cyclo (L-Prolyl-L-tyrosine), Cyclo(Pro-Phe), Emodin, Epiberberine, Syringetin, Tangeretin (4′,5,6,7,8-Pentamethoxyflavone)*, and Urolithin B—may spontaneously form stable complexes with the three core targets, with hydrogen bonding serving as a primary interaction force.

### 3.4. Analysis of Relative Metabolite Content

**Figure 8:**
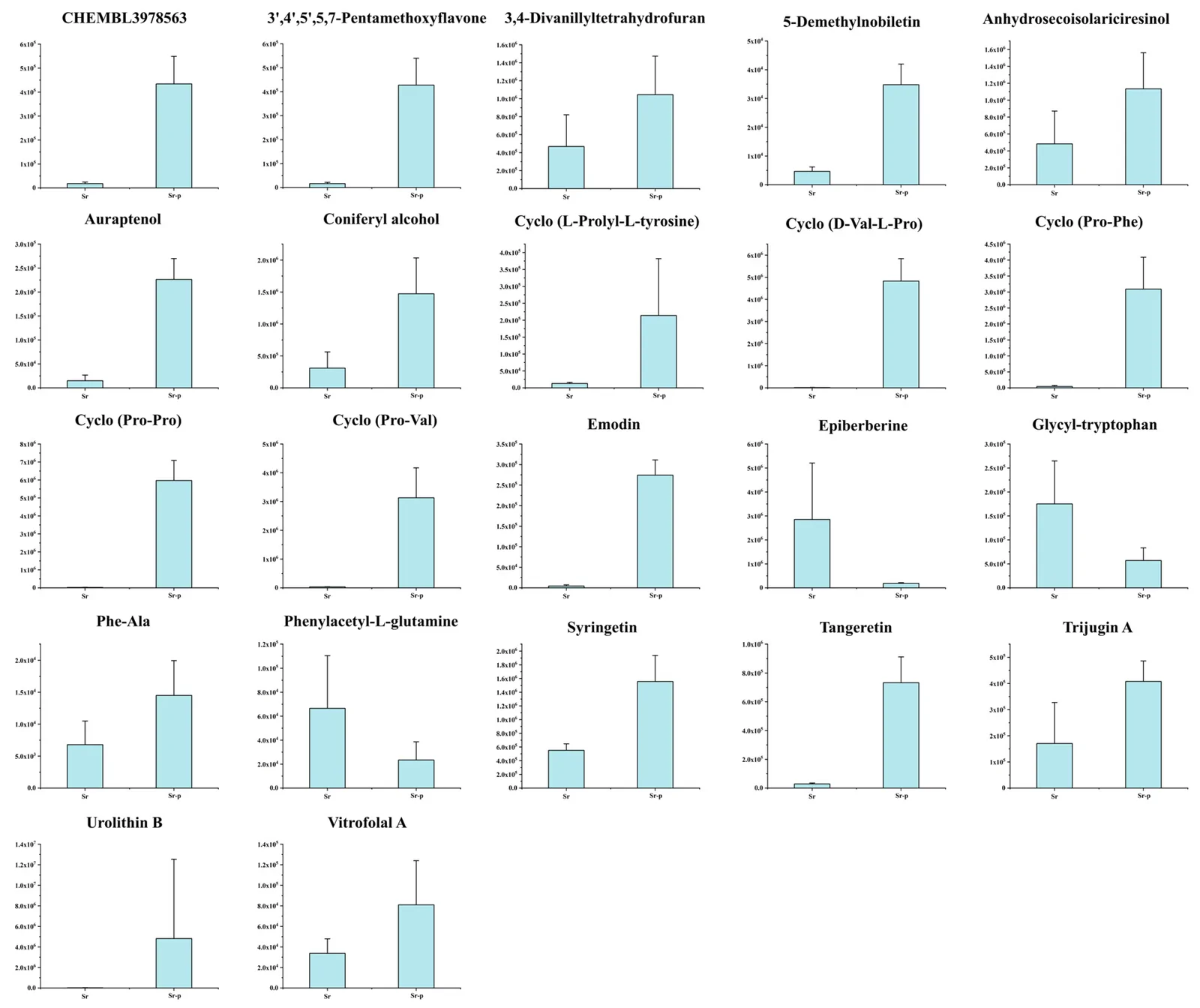
The relative abundance (peak area) of 22 differential metabolites with potential pharmacological activities at different processing stages of Euryales Semen. Error bars represent the standard deviation.

The relative contents of the 22 key metabolites were quantitatively compared before and after processing (Figure 8). The stir-frying process significantly upregulated 19 metabolites, including CHEMBL3978563, the cyclic dipeptides (e.g., Cyclo (L-Pro-L-Tyr), Cyclo (Pro-Phe)), PMFs (e.g., Tangeretin, Syringetin), and Urolithin B. Notably, Urolithin B was consistently detected in all raw samples with peak intensities markedly above baseline, while its levels in the processed samples increased by 168-fold, indicating a pronounced processing-induced elevation. In contrast, the contents of three metabolites, Glycyl-tryptophan, Phenylacetyl-L-glutamine, and Epiberberine, were downregulated, with Epiberberine showing a 93.47% reduction.

## 4. Discussion

Wheat bran stir-frying is a pivotal processing method that fundamentally alters the clinical application of Euryales Semen, shifting its focus from “securing essence” to “tonifying the spleen and kidney” [4]. Our integrated metabolomics and network pharmacology approach provides a systematic molecular narrative for this traditional wisdom. The core of this transformation lies in the significant reshaping of the metabolite profile, characterized by the upregulation of a cohort of key bioactive metabolites and their synergistic interactions with a network of disease-relevant targets.

The most striking change observed was the dramatic 168-fold increase in Urolithin B. This finding is particularly noteworthy because urolithins are typically gut microbiota-derived metabolites of ellagitannins and are seldom detected in plant tissues themselves [33]. The identification of Urolithin B was confirmed at Level 1 confidence using an authentic standard for retention time and MS/MS spectral matching. The identification of Urolithin B was confirmed at Level 1 confidence using an authentic standard for retention time and MS/MS spectral matching. Nonetheless, the detection of Urolithin B following bran stir-frying is consistent with a potential thermal transformation pathway from ellagitannin/ellagic acid precursors endogenous to E. ferox, although the precise mechanism remains to be fully elucidated. We therefore frame this as a hypothesis rather than a demonstrated conclusion, and future studies employing thermal simulation experiments, intermediate product tracking, and additional standard-based confirmations are warranted to validate this proposed pathway.

It should be noted that while stir-frying at 200 °C inevitably drives off moisture, all metabolite quantifications were normalized to the dry weight of lyophilized powder (Section 2.3.1), ensuring that the observed increases cannot be attributed solely to concentration effects from mass loss. Among the 19 upregulated metabolites, those showing moderate fold changes (e.g., 2- to 5-fold increases) may partially reflect such concentration effects. However, metabolites like Urolithin B (168-fold), Emodin (62-fold), and 3-(Hydroxymethyl)phenol (87-fold) exhibit increases far exceeding what could be explained by water loss alone. Moreover, Urolithin B was undetectable in raw samples and emerged prominently after processing, supporting a processing-induced transformation rather than a passive concentration artifact.

Critically, the multifaceted biological activities reported for Urolithin B—including anti-aging, ameliorating cognitive deficits [34], anti-atherosclerotic [35], improving insulin sensitivity [36,37], and anti-tumor effects [38,39,40]—exhibit a remarkable convergence with the documented pharmacological profile of Euryales Semen [5,41]. Therefore, the surge in Urolithin B content may contribute to the broad-spectrum efficacy enhancement of processed Euryales Semen, particularly its strengthened tonifying and chronic disease-preventive effects. Our molecular docking results further suggest a potential role by revealing predicted strong binding affinities to core targets like MMP2 and MMP9, which are intimately associated with aging and cancer pathways, although these computational predictions require experimental verification.

From the perspective of traditional Chinese medicine (TCM), the selection of MMP2 and MMP9 as core targets may offer a conceptual bridge to the “Spleen- and Kidney-tonifying” effects of processed Euryales Semen. MMP2 and MMP9 are key enzymes involved in extracellular matrix degradation, tissue remodeling, inflammation, and fibrosis—pathological processes that share conceptual parallels with the TCM concepts of “Spleen deficiency with dampness retention” and “Kidney deficiency with tissue degeneration”. It should be noted, however, that this correspondence is intended as an interpretive framework to contextualize our findings within TCM theory, rather than a claim of established mechanistic equivalence. The synergistic targeting of MMP2 and MMP9 by multiple upregulated metabolites also reflects the multi-component, multi-target characteristics of TCM holistic regulation.

Beyond Urolithin B, the processing also significantly elevated the levels of several polymethoxyflavones (PMFs), notably Tangeretin and Syringetin. Tangeretin has been extensively documented to possess anti-tumor activity by inducing cell cycle arrest and apoptosis, often through modulation of the PI3K/Akt/GSK3B pathway and PARP1 activation [42,43,44]. Similarly, Syringetin has demonstrated α-glucosidase inhibitory activity, relevant for diabetes management, and the ability to inhibit cancer cell proliferation [45,46]. The co-upregulation of these flavonoids suggests a multi-pronged enhancement of the anti-tumor and anti-diabetic potential of processed Euryales Semen. Our network pharmacology findings, which identified “neoplasms” as the disease node with the highest degree, strongly support this assertion.

The network analysis did not merely identify individual metabolites but revealed a collaborative network of 22 key metabolites. Among these, cyclic dipeptides such as Cyclo (L-Pro-L-Tyr) and Cyclo (Pro-Phe) deserve special attention. Their increased content post-processing is highly significant. Cyclo (L-Pro-L-Tyr) has been shown to exhibit tyrosinase inhibitory activity [47], directly aligning with the traditional use of Euryales Semen and its documented anti-melanogenic effects [48]. Concurrently, Cyclo (Pro-Phe) has demonstrated cytotoxicity against human colon cancer cells [49], further diversifying the anti-tumor arsenal of the processed herb. The presence of these potent peptides underscores the contribution of non-flavonoid components to the overall efficacy.

The true mechanistic insight from this study lies not in the action of a single component, but in the emergent synergistic network. The 22 key metabolites do not act in isolation; they converge on a limited set of 10 core targets, including MMP2, MMP9 and AKR1B1. Molecular docking predicted that multiple upregulated metabolites, including Urolithin B, Tangeretin, and Syringetin, may bind strongly to these same targets, providing a hypothesis-generating basis for future experimental studies. For instance, both Tangeretin and Urolithin B were predicted to show high affinity for MMP2 and MMP9. This computational prediction suggests that the enhanced efficacy of processed Euryales Semen might be partially attributed to the collective, synergistic action of multiple upregulated metabolites, which co-regulate key signaling hubs involved in metabolism, cell proliferation, and inflammation, although this hypothesis awaits experimental validation. This multi-component, multi-target network effect provides a sophisticated scientific rationale for the holistic action of processed Euryales Semen, perfectly echoing the core principles of TCM. To contextualize our docking results against known ligands, we compared the predicted binding affinities of key metabolites with literature-reported data for MMP2 and MMP9 inhibitors. For MMP9, Urolithin B has been reported to exhibit in vitro inhibitory activity against recombinant human MMP-9 with an IC_50_ of 13.17 µM, with surface plasmon resonance (SPR) confirming direct binding (K_D = 4.3 × 10^−5^ M); molecular docking in the same study predicted a binding energy of −8.54 kcal·mol^−1^ for Urolithin B with MMP9 (PDB ID: 1L6J) [50]. Our docking result for Urolithin B with MMP9 (−9.38 kcal·mol^−1^) is comparable to this reported value. Similarly, emodin—another top MMP9-targeting metabolite identified in our study—has been reported to inhibit MMP-9 activity with an IC_50_ of 15 µM [51]. Furthermore, an independent molecular docking study predicted a binding energy of −8.702 kcal·mol^−1^ for the emodin–MMP9 interaction [52], which is in good agreement with our predicted binding affinity of −9.20 kcal·mol^−1^ for this pair. This consistency further supports the biological relevance of our docking predictions. For MMP2, SB3CT is a well-known mechanism-based inhibitor widely used as a positive control in docking studies [53,54]. The binding energies of our top MMP2-targeting metabolites (e.g., 3′,4′,5′,5,7-Pentamethoxyflavone at −9.87 kcal·mol^−1^; 5-Demethylnobiletin at −9.79 kcal·mol^−1^) are within a range comparable to those reported for known MMP2 inhibitors, further supporting the biological relevance of these predicted interactions. It should be noted that the binding energy threshold of −6.0 kcal·mol^−1^ used in this study was selected as a reference cutoff to facilitate comparison with previously reported docking studies. While polyphenolic and flavonoid ligands tend to exhibit more negative binding energies due to their larger molecular size and greater number of rotatable bonds, our ligand efficiency analysis (presented in Section 3.3) confirmed that the strong binding affinities of the upregulated metabolites are not merely a size artifact. All top hits exhibited LE values below −0.3 kcal·mol^−1^ per heavy atom, suggesting energetically favorable interactions with their respective targets.

Conversely, the significant decrease (93.47%) in the content of Epiberberine is equally instructive. While this alkaloid possesses bioactivities, it is also associated with potential gastrointestinal irritation and immunotoxicity at high concentrations [55,56]. As a representative isoquinoline alkaloid derived from Coptidis Rhizoma, Epiberberine shares structural and biosynthetic characteristics with other major Coptis alkaloids, and its toxicity profile is consistent with the known safety concerns associated with Coptis alkaloids. Although direct toxicological data specifically for Epiberberine remain limited, the observed reduction in this component during stir-frying aligns with the traditional purpose of processing to ‘modify drug properties’ and may be interpreted as a potential contributing factor to the mitigation of gastrointestinal side effects, potentially making the medicine gentler for spleen and stomach tonification. We acknowledge that this interpretation remains speculative in the absence of direct toxicological data for Epiberberine itself and therefore frame it as a hypothesis that requires dedicated experimental verification.

**Limitations and Future Perspectives:** While our integrated approach provides robust predictions, it is important to acknowledge the limitations of in silico analyses. The network pharmacology and molecular docking results are predictive and require further experimental validation. Future research should focus on in vitro and in vivo studies to confirm the biological activities of the identified key metabolites (especially Urolithin B) and their interactions with the core targets (e.g., MMP2, MMP9). Additionally, elucidating the precise thermal degradation pathways that lead to the formation of Urolithin B during processing would be a valuable direction for future investigation. Furthermore, metabolomics does not capture all possible changes; other omics techniques like proteomics could provide complementary insights.

## 5. Conclusions

This study offers a comprehensive characterization of the efficacy transformation of Euryales Semen after wheat bran stir-frying by employing an integrated metabolomics, network pharmacology, and molecular docking strategy. Beyond merely cataloguing metabolic changes, our work provides three key insights: First, we observed that processing acts as a chemical reactor, significantly upregulating a suite of bioactive metabolites, with the dramatic emergence of Urolithin B being particularly remarkable. Second, we constructed a “metabolite-target-disease” network, pinpointing key compounds like Tangeretin, Syringetin, and 5-Demethylnobiletin, and core targets such as MMP2, MMP9 and AKR1B1, which may collaboratively contribute to the enhanced tonifying, anti-tumor, and anti-diabetic properties. Third, molecular docking predicted strong binding of these key metabolites to the core targets, thereby offering a computational basis for the network pharmacology predictions that warrants further experimental investigation.

Our findings link traditional processing theory with modern scientific evidence, providing a preliminary computational basis that may serve as a useful reference for the quality control and optimized clinical application of processed Euryales Semen. The tentative identification of processing-induced metabolites such as Urolithin B offers a potential starting point for investigating the biosynthetic pathways activated by traditional pharmaceutical techniques, although this remains a hypothesis that requires rigorous experimental verification. Future work should focus on the in vivo validation of these predicted targets and pathways and further explore the precise reconstruction mechanisms of the metabolic network during the stir-frying process.

## Figures and Tables

**Table 1:** Parameters of gradient elution.

Time/min	Mobile Phase A%	Mobile Phase B%
0	95	5
9	5	95
10	5	95
11.1	95	5
14	95	5

**Table 2:** The classification of 244 differential metabolites.

Class	Sr vs. Sr-p	
	Down	Up
Alkaloids	3	16
Amino acids and derivatives	6	16
Flavonoids	6	40
Lignans and Coumarins	1	22
Lipids	8	10
Nucleotides and derivatives	0	11
Organic acids	2	22
Others	1	25
Phenolic acids	7	40
Quinones	0	2
Tannins	0	1
Terpenoids	0	5
Total	244	

Note: up/down indicates that the metabolite content of the latter group has a changing trend compared with the previous group.

**Table 3:** Summary of molecular docking results for key bioactive metabolites identified from metabolomics analysis.

Key Bioactive Metabolites	Binding Energy/(kcal·mol^−1^)									
	AKR1B1 (8FH8)	AKT1 (8UW9)	ALOX5 (6N2W)	GSK3B (9X2Q)	IGF1R (8PYN)	MMP2 (7XJO)	MMP9 (8K5Y)	PARP1 (7ONS)	PIK3CG (6AUD)	PTGS2 (5F19)
[(1R,2S)-1-(1,3-benzodioxol-5-yl)-2-methyl-3-oxobutyl]4-hydroxy-3-methoxybenzoate *	−7.86	−6.27	−6.10	−5.39	−7.04	−8.67	−6.55	−6.69	−5.67	−6.91
3′,4′,5′,5,7-Pentamethoxyflavone *	−8.09	−6.59	−5.32	−5.99	−6.83	−9.87	−6.29	−6.24	−6.81	−7.11
3,4-Divanillyltetrahydrofuran *	−6.80	−5.98	−7.14	−6.20	−5.73	−9.46	−6.91	−5.78	−5.99	−5.85
5-Demethylnobiletin; 5-Hydroxy-6,7,8,3′,4′-Pentamethoxyflavone *	−6.71	−6.72	−4.82	−5.97	−6.93	−9.79	−6.09	−5.80	−6.14	−7.76
Anhydrosecoisolariciresinol (AHS) *	−7.57	−6.62	−4.99	−6.38	−6.70	−8.36	−6.24	−6.03	−6.03	−6.05
Auraptenol	−7.15	−6.45	−5.80	−6.10	−6.49	−7.83	−5.58	−5.50	−5.85	−6.21
Coniferyl alcohol	−6.11	−5.37	−5.29	−5.34	−6.31	−6.42	−6.79	−5.57	−5.62	−5.18
Cyclo (L-Prolyl-L-tyrosine)	−7.30	−7.54	−7.10	−7.89	−7.98	−8.59	−9.30	−6.26	−7.05	−7.75
Cyclo(D-Val-L-Pro)	−6.95	−5.79	−6.11	−5.72	−5.59	−6.85	−7.70	−5.73	−5.51	−6.11
Cyclo(Pro-Phe)	−7.28	−7.28	−6.64	−7.27	−7.94	−8.27	−9.26	−6.06	−6.96	−7.34
Cyclo(Pro-Pro)	−6.40	−6.21	−6.37	−6.16	−6.03	−7.02	−7.16	−5.31	−5.90	−6.12
Cyclo(Pro-Val)	−5.99	−6.24	−6.15	−6.23	−6.06	−7.15	−7.69	−5.46	−5.66	−5.90
Emodin	−7.18	−7.06	−6.21	−7.06	−8.45	−8.99	−9.20	−6.99	−7.55	−7.72
Epiberberine	−7.32	−7.37	−7.28	−6.96	−7.59	−9.64	−7.32	−6.47	−7.36	−8.72
Glycyl-tryptophan	−6.94	−7.77	−6.19	−7.97	−7.32	−8.34	−7.40	−5.91	−5.90	−6.45
Phe-Ala	−5.96	−7.04	−4.80	−6.18	−5.59	−7.37	−7.47	−5.76	−6.22	−6.18
Phenylacetyl-L-glutamine	−6.18	−6.10	−4.84	−6.88	−6.00	−7.13	−7.64	−4.92	−5.94	−5.86
Syringetin	−7.16	−6.61	−6.70	−6.30	−7.66	−9.42	−7.08	−7.06	−6.37	−7.32
Tangeretin (4′,5,6,7,8-Pentamethoxyflavone) *	−7.89	−5.85	−6.02	−7.13	−6.51	−9.09	−5.79	−6.19	−6.16	−7.43
Trijugin A	−8.36	−6.94	−5.03	−6.36	−6.66	−7.75	−6.44	−6.19	−6.44	−6.11
Urolithin B	−7.77	−7.29	−7.54	−6.97	−7.83	−8.00	−9.38	−6.62	−6.83	−6.55
Vitrofolal A	−7.63	−6.69	−5.72	−5.97	−7.75	−8.16	−6.18	−6.73	−7.22	−7.75

## Data Availability

The raw MS data supporting the conclusions of this article are available at the NIH Common Fund’s National Metabolomics Data Repository (NMDR) website, the Metabolomics Workbench [57], https://www.metabolomicsworkbench.org, where they have been assigned Project ID PR003331. The data can be accessed directly via the Project DOI: 10.21228/M8428V. The data will be publicly available on 29 August 2027. The minimal dataset supporting the main conclusions has also been provided as Supplementary Materials. Requests for additional data can be directed to the corresponding author.

## Supplementary Materials

The following supporting information can be downloaded at: https://www.mdpi.com/article/10.3390/metabo16090669/s1, Figure S1: Intersection target; Table S1: Information on differential metabolites of *Euryale ferox* that have potential physiological activity; Table S2: Information on key metabolites of Euryale ferox that have potential physiological activity (with *p*-values); Table S3: Re-docking RMSD results for all ten target proteins.

## Abbreviations

The following abbreviations are used in this manuscript:

UPLC-MS/MS	Ultra-Performance Liquid Chromatography–Tandem Mass Spectrometry
ESI	Electrospray Ionization
MRM	Multiple Reaction Monitoring
QC	Quality Control
DP	Declustering Potential
CE	Collision Energy
PCA	Principal Component Analysis
OPLS-DA	Orthogonal Partial Least Squares–Discriminant Analysis
VIP	Variable Importance in Projection
FC	Fold Change
PPI	Protein–Protein Interaction
SMILES	Simplified Molecular-Input Line-Entry System
PDB	Protein Data Bank
PMFs	Polymethoxyflavones
TCM	Traditional Chinese Medicine
AKR1B1	Aldo-Keto Reductase Member B1
AKT1	AKT Serine/Threonine Kinase 1
ALOX5	Arachidonate 5-Lipoxygenase
GSK3B	Glycogen Synthase Kinase 3 Beta
IGF1R	Insulin-like Growth Factor 1 Receptor
MMP2	Matrix Metallopeptidase 2
MMP9	Matrix Metallopeptidase 9
PARP1	Poly(ADP-Ribose) Polymerase 1
PIK3CG	Phosphatidylinositol-4,5-Bisphosphate 3-Kinase Catalytic Subunit Gamma
PTGS2	Prostaglandin-Endoperoxide Synthase 2
sr	Raw (unprocessed) *Euryale ferox* seed
sr-p	Processed *Euryale ferox* seed
