# Supplementary material for: Wheat Bran Stir-Frying Reshapes the Metabolome of Euryale Ferox Seeds and Predicts Enhanced Bioactive Potential for Spleen and Kidney Tonifying
Source: Metabolites. 2026 Sep 10;16(9):669. doi: 10.3390/metabo16090669 (PMC13609870; doi:10.3390/metabo16090669)
Supplement: Supplementary file 1 [file metabolites-16-00669-s001.zip › Table S1.pdf]

Table S1. Information on differential metabolites of *Euryale ferox* that have potential physiological activity.

Compounds	Class I	Class II	Type
(3-(carboxyamino)-2-methylpropanoyl)phenylalanine	Amino acids and derivatives	Amino acids and derivatives	up
(3S)-1,3-dihydroxy-2,3,3a,4-tetrahydropyrrolo[2,1-b]quinazolin-9(1H)-one	Alkaloids	Alkaloids	up
(7'R)-7'-Hydroxylariciresinol	Others	Others	up
(7S,8R-Threo-3',4,9'-trihydroxy-3-methoxy-4',7-epoxy-neolignan-9-O-rhamnoside	Lignans and Coumarins	Lignans	down
(E)-4-Hydroxyhex-2-enoic acid	Organic acids	Organic acids	up
(R)-3-ethyl-7-hydroxy-6-methoxyphthalide	Others	Others	up
(S)-Peucedanol	Lignans and Coumarins	Coumarins	up
[(1R,2S)-1-(1,3-benzodioxol-5-yl)-2-methyl-3-oxobutyl]4-hydroxy-3-methoxybenzoate	Lignans and Coumarins	Lignans	up
1,3,6,7-tetrahydroxy-2-(3,4,5-trihydroxyoxan-2-yl)xanthen-9-one	Flavonoids	Other Flavonoids	up
12-Methyltetradecanoic Acid	Lipids	Free fatty acids	down
13-KODE	Lipids	Free fatty acids	up
15(R)-Hydroxylinoleic Acid	Lipids	Free fatty acids	up

1-Acetyl-β-carboline	Alkaloids	Plumerane	up
1-Methylxanthine	Nucleotides and derivatives	Nucleotides and derivatives	up
1-O-Caffeoyl-β-D-glucose	Phenolic acids	Phenolic acids	down
2,2'-Dihydroxybiphenyl	Phenolic acids	Phenolic acids	up
2,4-Dihydroxybenzaldehyde	Others	Aldehyde compounds	up
2,5-Dihydroxyacetophenone	Others	Ketone compounds	up
2-aminodocoSane-1,6,19,20,21-pentaol	Lipids	Sphingolipids	up
2-Aminoethylphosphonate	Others	Others	up
2-Aminohexadecane-1,5,15-triol	Lipids	Sphingolipids	down
2-Aminotetradecane-1,11,13-triol	Lipids	Sphingolipids	down
2-Aminotetradecane-1,4-diol	Lipids	Sphingolipids	down
2-Aminotetradecane-1,5,13-triol	Lipids	Sphingolipids	down
2-Furoic acid	Organic acids	Organic acids	up
2-Hydroxy-2-methyl-3-oxobutanoic acid	Organic acids	Organic acids	up

2'-Hydroxy-4'-Methylacetophenone	Phenolic acids	Phenolic acids	up
2-Hydroxybutyric Acid	Organic acids	Organic acids	up
2-Hydroxyisocaproic acid	Organic acids	Organic acids	up
2-hydroxynaringenin	Flavonoids	Flavanones	up
2-Hydroxyphenylacetic acid	Organic acids	Organic acids	up
2-Methoxycinnamic acid	Phenolic acids	Phenolic acids	up
2-Methylsuccinic acid	Organic acids	Organic acids	up
2-Picolinic acid	Organic acids	Organic acids	up
3-(Hydroxymethyl)phenol	Phenolic acids	Phenolic acids	up
3,3',5,7-Tetrahydroxy-4',6-Dimethoxyflavone	Flavonoids	Flavonols	up
3',4',5',5,7-Pentamethoxyflavone	Flavonoids	Flavones	up
3,4-Dihydrocoumarin	Lignans and Coumarins	Coumarins	up
3,4-Dihydroxybenzoic Acid Ethyl Ester	Phenolic acids	Phenolic acids	up
3,4-Dimethoxyphenyl acetic acid	Phenolic acids	Phenolic acids	up

3,4-Divanillyltetrahydrofuran	Phenolic acids	Phenolic acids	up
3,4-Methylenedioxy cinnamyl alcohol	Others	Alcohol compounds	up
3,7-Di-O-methylquercetin	Flavonoids	Flavonols	up
3-[(1-Carboxyvinyl)oxy]benzoic acid	Phenolic acids	Phenolic acids	up
3-Hydroxy-1-(4-hydroxy-3,5-dimethoxyphenyl)propan-1-one	Others	Ketone compounds	up
3-Hydroxy-3-methylpentane-1,5-dioic acid	Amino acids and derivatives	Amino acids and derivatives	up
3-Hydroxycycloarta-24-ene-26-oic acid	Terpenoids	Triterpene	up
3-Hydroxyphenylacetic Acid Methyl Ester	Phenolic acids	Phenolic acids	up
3-Hydroxypropanoic acid	Organic acids	Organic acids	up
3-Hydroxypyridine	Alkaloids	Pyridine alkaloids	up
3'-linoleoyl sucrose	Lipids	Free fatty acids	down
3-Methyl-2-Oxobutanoic acid	Organic acids	Organic acids	up
3-Methyl-2-oxopentanoic acid	Organic acids	Organic acids	up
3-Methylxanthine	Nucleotides and derivatives	Nucleotides and derivatives	up

3-Oleoyl sucrose	Lipids	Free fatty acids	down
4-Allylcatechol	Phenolic acids	Phenolic acids	up
4-Aminobenzoic acid	Phenolic acids	Phenolic acids	up
4-Aminophenol	Alkaloids	Phenolamine	up
4-Hydroxy-2-Oxopentanoic Acid	Organic acids	Organic acids	up
4-Hydroxy-3,5-dimethoxybenzyl alcohol	Phenolic acids	Phenolic acids	up
4'-Hydroxy-3'-methoxyacetophenone	Others	Ketone compounds	up
4-Hydroxyacetophenone	Others	Ketone compounds	up
4-Hydroxybenzaldehyde	Others	Aldehyde compounds	up
4-Hydroxycoumarin	Lignans and Coumarins	Coumarins	up
4-Hydroxycyclohexylcarboxylic acid	Organic acids	Organic acids	up
4-Hydroxyquinoline	Alkaloids	Quinoline alkaloids	up
4-Methoxycinnamic acid	Phenolic acids	Phenolic acids	up
4-Nitrocatechol	Phenolic acids	Phenolic acids	up

4-O-Glucosyl-sinapate	Phenolic acids	Phenolic acids	down
4-Oxopentanoic Acid	Organic acids	Organic acids	up
5,6-Dihydro-5-methyluracil	Nucleotides and derivatives	Nucleotides and derivatives	up
5,7,3',4',5'-Pentahydroxydihydroflavone	Flavonoids	Flavanones	up
5-Demethylnobiletin	Flavonoids	Flavones	up
5-Hydroxy-3,3',4',5',7-Pentamethoxyflavone	Flavonoids	Flavones	up
5-Hydroxymethylfurfural	Others	Aldehyde compounds	up
5-Hydroxyquinoline	Alkaloids	Quinoline alkaloids	up
5-Methylcytosine	Nucleotides and derivatives	Nucleotides and derivatives	up
5-O-Caffeoylshikimic acid	Phenolic acids	Phenolic acids	up
5-Ribofuranosylnicotinamide	Nucleotides and derivatives	Nucleotides and derivatives	up
6,7-Dihydroxy-4-methylcoumarin	Lignans and Coumarins	Coumarins	up
6,8-Dihydroxy-3-methylisocoumarin	Lignans and Coumarins	Coumarins	up

6-C-MethylKaempferol-3-glucoside	Flavonoids	Flavones	up
6-Hydroxyhexanoic acid	Organic acids	Organic acids	up
6-Hydroxynicotinic acid	Alkaloids	Pyridine alkaloids	up
7-Hydroxy-4-chromone	Others	Chromone	up
7-Methylxanthine	Nucleotides and derivatives	Nucleotides and derivatives	up
7-O-Galloyltricetiflavan	Flavonoids	Flavanols	up
8-Azaguanine	Nucleotides and derivatives	Nucleotides and derivatives	up
9,10-dimethoxy-5-(3,4,5-trimethoxyphenyl)furo[3',4':6,7]naphtho[2,3-d][1,3]dioxol-6(8H)-one	Lignans and Coumarins	Lignans	up
9-Hydroxy-10,12,15-octadecatrienoic acid	Lipids	Free fatty acids	up
9-Hydroxy-12-oxo-10(E),15(Z)-octadecadienoic acid	Lipids	Free fatty acids	up
9-Hydroxy-12-oxo-15(Z)-octadecenoic acid	Lipids	Free fatty acids	up
9s,13r-12-Oxophytodienoic Acid	Lipids	Free fatty acids	up
Abrusin	Flavonoids	Flavones	down
Abscisic acid	Organic acids	Organic acids	up

Acacetin-7-O-rutinoside	Flavonoids	Flavones	down
Acetoxyacetic acid	Organic acids	Organic acids	up
Afzelechin	Flavonoids	Flavanols	down
Anhydrosecoisolariciresinol	Lignans and Coumarins	Lignans	up
Arachidic acid	Lipids	Free fatty acids	down
Aromadendrin	Flavonoids	Flavanonols	up
Auraptenol	Lignans and Coumarins	Coumarins	up
Axillarin	Flavonoids	Flavonols	up
Brevifolin	Phenolic acids	Phenolic acids	down
Cagayanone A	Lignans and Coumarins	Lignans	up
Capillarisin	Others	Chromone	up
Chrysoeriol-6-C-rhamnoside-7-O-rhamnoside	Flavonoids	Flavones	down
Cimicifugamide A	Alkaloids	Phenolamine	down
Coniferaldehyde	Others	Aldehyde compounds	up

Coniferin	Phenolic acids	Phenolic acids	down
Coniferyl alcohol	Phenolic acids	Phenolic acids	up
Cyclo (L-Prolyl-L-tyrosine	Amino acids and derivatives	Amino acids and derivatives	up
Cyclo(D-Val-L-Pro)	Amino acids and derivatives	Amino acids and derivatives	up
Cyclo(Pro-Glu)	Amino acids and derivatives	Amino acids and derivatives	up
Cyclo(Pro-Phe)	Amino acids and derivatives	Amino acids and derivatives	up
Cyclo(Pro-Pro)	Amino acids and derivatives	Amino acids and derivatives	up
Cyclo(Pro-Val)	Amino acids and derivatives	Amino acids and derivatives	up
Cyclo(Val-Ala)	Amino acids and derivatives	Amino acids and derivatives	up
D-Arabinose	Others	Saccharides	up
Dehydrovomifoliol	Terpenoids	Monoterpenoids	up
Delta-Tocopherol	Others	Vitamin	up
demethoxypinoresinol	Lignans and Coumarins	Lignans	up

Demethyl-dehydroxysecoisolariciresinol	Lignans and Coumarins	Lignans	up
Dihydrodehydrodiconiferyl alcohol-4-O-glucoside	Lignans and Coumarins	Lignans	up
Dihydromyricetin	Flavonoids	Flavanonols	up
Dihydrophaseic acid	Terpenoids	Sesquiterpenoids	up
Diosmetin-7-O-galactoside	Flavonoids	Flavones	up
Diosmetin-7-O-Neohesperidoside	Flavonoids	Flavones	up
Dopamine	Alkaloids	Phenolamine	up
D-Panthenol	Others	Alcohol compounds	up
Emodin	Quinones	Anthraquinone	up
Epiberberine	Alkaloids	Isoquinoline alkaloids	down
Epigallocatechin-(4β->8)-epigallocatechin-3-O-gallate	Flavonoids	Flavanols	up
Ethyl maltol	Phenolic acids	Phenolic acids	up
Ethylmalonic acid	Organic acids	Organic acids	up
Ferulic β-glucoside	Phenolic acids	Phenolic acids	down

Fortuneanoside A	Phenolic acids	Phenolic acids	up
Fraxetin	Lignans and Coumarins	Coumarins	up
Gallic Acid Ethyl Ester	Phenolic acids	Phenolic acids	up
Gemin D	Tannins	Tannin	up
gentisic acid 5-O-β-D-(6'-O-galloyl)-gluco-pyranoside	Phenolic acids	Phenolic acids	up
Glycyl-tryptophan	Amino acids and derivatives	Amino acids and derivatives	down
Gly-Glu-Met	Amino acids and derivatives	Amino acids and derivatives	down
Hexanoyl-L-glycine	Amino acids and derivatives	Amino acids and derivatives	up
h-gamma-Glu-leu-oh	Amino acids and derivatives	Amino acids and derivatives	up
Hispidulin-7-O-glucoside	Flavonoids	Flavones	up
Hydrangeifolin I	Phenolic acids	Phenolic acids	up
Hydroxy ricinoleic acid	Lipids	Free fatty acids	up
Icariside E4	Lignans and Coumarins	Lignans	up
Indole-3-carboxylic acid	Alkaloids	Plumerane	up

Indole-5-carboxylic acid	Alkaloids	Plumerane	up
Iristectorin A	Flavonoids	Isoflavones	up
Isocytosine	Nucleotides and derivatives	Nucleotides and derivatives	up
isoemodin	Quinones	Anthraquinone	up
Isohyperoside	Flavonoids	Flavonols	up
Isonicotinic acid	Others	Vitamin	up
Isosalipurposide	Flavonoids	Chalcones	up
JiangxiBaiyingsu I	Terpenoids	Sesquiterpenoids	up
Justicidin A	Lignans and Coumarins	Lignans	up
Justicidin C	Lignans and Coumarins	Lignans	up
L-Carnitine	Alkaloids	Alkaloids	down
ligballinol	Lignans and Coumarins	Lignans	up
Limocitrin	Flavonoids	Flavonols	up
Limocitrin 3-Glucoside	Flavonoids	Flavones	up

Limocitrin-3-O-glucoside	Flavonoids	Flavonols	down
L-Isoleucyl-L-Aspartate	Amino acids and derivatives	Amino acids and derivatives	down
L-Tartaric acid	Organic acids	Organic acids	up
L-Theanine	Amino acids and derivatives	Amino acids and derivatives	down
Lumazine	Nucleotides and derivatives	Nucleotides and derivatives	up
Luteolin-6-C-glucoside	Flavonoids	Flavones	up
L-Xylose	Others	Saccharides	up
LysoPC 19:2	Lipids	LPC	up
Maesopsin	Flavonoids	Other Flavonoids	up
Meranzin	Lignans and Coumarins	Coumarins	up
Methyl 2-O-(4-hydroxybenzoyl)-2,4,6-trihydroxyphenylacetate	Phenolic acids	Phenolic acids	up
Methyl 4-hydroxybenzoate	Phenolic acids	Phenolic acids	up
Methyl linolenate	Lipids	Free fatty acids	up
Methyl Syringate	Phenolic acids	Phenolic acids	up

Myricetin-3-O-(6"-acetyl)glucoside	Flavonoids	Flavonols	up
N-(1-deoxy-1-xylosyl)valine	Amino acids and derivatives	Amino acids and derivatives	down
N,N-Dimethyl-5-methoxytryptamine	Alkaloids	Plumerane	up
N-Acetyl-L-leucine	Amino acids and derivatives	Amino acids and derivatives	up
Nectandrin B	Lignans and Coumarins	Lignans	up
N-Ethylmaleimide	Amino acids and derivatives	Amino acids and derivatives	up
Nicotinamide	Others	Vitamin	up
Nicotinate D-ribonucleoside	Others	Vitamin	down
Nicotinic acid	Others	Vitamin	up
Nicotinic Acid Methyl Ester	Alkaloids	Pyridine alkaloids	up
Nobiletin	Flavonoids	Flavones	up
Norharmane	Alkaloids	Plumerane	up
Okanin-4'-(6"-O-acetyl)glucoside	Flavonoids	Chalcones	up
Okanin-4'-O-(6"-O-malonyl)glucoside	Flavonoids	Chalcones	up

Orotidine	Others	Vitamin	up
Patriscabratine	Alkaloids	Alkaloids	up
p-Coumaraldehyde	Phenolic acids	Phenolic acids	down
p-Coumaric acid methyl ester	Phenolic acids	Phenolic acids	up
p-Coumaroylmalic acid	Phenolic acids	Phenolic acids	up
Phe-Ala	Amino acids and derivatives	Amino acids and derivatives	up
Phenyl acetate	Phenolic acids	Phenolic acids	up
Phenylacetyl-L-glutamine	Amino acids and derivatives	Amino acids and derivatives	down
Phloroglucinol	Phenolic acids	Phenolic acids	up
Physakengose P	Phenolic acids	Phenolic acids	up
Prolylhydroxyproline	Amino acids and derivatives	Amino acids and derivatives	up
propyl-L-alanine	Amino acids and derivatives	Amino acids and derivatives	up
Protocatechuic Acid Methyl Ester	Phenolic acids	Phenolic acids	up
Prunetin	Flavonoids	Isoflavones	up

Pyrocatechol	Phenolic acids	Phenolic acids	up
Pyrogallol	Phenolic acids	Phenolic acids	up
Quercetagetin-3,4'-Dimethyl Ether	Flavonoids	Flavonols	up
Quercetagetin-3-Methyl Ether	Flavonoids	Flavonols	up
Quercetagetin-4'-Methyl Ether	Flavonoids	Flavonols	up
Quercetin-3-O-arabinoside	Flavonoids	Flavonols	up
Riboflavin	Others	Vitamin	up
Robinetin	Flavonoids	Flavonols	up
Robinson-7-O-Neohesperidin	Flavonoids	Flavonols	down
Rosmarinic acid	Phenolic acids	Phenolic acids	down
Salicylic acid	Phenolic acids	Phenolic acids	up
Salireposide	Phenolic acids	Phenolic acids	up
Sclerophynin A	Phenolic acids	Phenolic acids	up
Shikimic acid	Organic acids	Organic acids	down

Shikimic Acid-3-Phosphate	Organic acids	Organic acids	up
Sinapyl alcohol	Phenolic acids	Phenolic acids	up
Skimmin	Lignans and Coumarins	Coumarins	up
Suberic Acid	Organic acids	Organic acids	up
Synephrine	Alkaloids	Phenolamine	up
Syringaldehyde	Others	Aldehyde compounds	up
Syringetin	Flavonoids	Flavonols	up
Syringetin-3-O-glucoside	Flavonoids	Flavonols	up
Syringic acid	Phenolic acids	Phenolic acids	up
Syringoyl-D-Glucose	Phenolic acids	Phenolic acids	up
Tamarixetin	Flavonoids	Flavonols	up
Tangeretin	Flavonoids	Flavones	up
Theasinensin B	Flavonoids	Flavanols	up
Thymine	Nucleotides and derivatives	Nucleotides and derivatives	up

Tricin-4'-O-glucoside	Flavonoids	Flavones	up
Trijugin A	Terpenoids	Sesquiterpenoids	up
Tryptophol	Alkaloids	Plumerane	up
Tyrosol	Phenolic acids	Phenolic acids	up
Uridine 5'-diphospho-D-glucose	Nucleotides and derivatives	Nucleotides and derivatives	up
Urolithin B	Phenolic acids	Phenolic acids	up
Vanillin	Others	Aldehyde compounds	up
Vitrofolal A	Lignans and Coumarins	Lignans	up
α-Ketoglutaric acid	Organic acids	Organic acids	up
γ-Aminobutyric acid	Organic acids	Organic acids	down
