# Supplementary material for: Wheat Bran Stir-Frying Reshapes the Metabolome of Euryale Ferox Seeds and Predicts Enhanced Bioactive Potential for Spleen and Kidney Tonifying
Source: Metabolites. 2026 Sep 10;16(9):669. doi: 10.3390/metabo16090669 (PMC13609870; doi:10.3390/metabo16090669)
Supplement: Supplementary file 1 [file metabolites-16-00669-s001.zip › Table S2.pdf]

Table S2. Information on key metabolites of Euryale ferox that have potential physiological activity (with p-values)

Name	The number of associated targets	Potential physiological activity	P-value
Tangeretin	148	Neoplasms, Hypertension, Diabetes Mellitus, Atherosclerosis, Kidney Diseases	0.0209902427609385
5-Demethylnobiletin	146	Neoplasms, Hypertension, Atherosclerosis, Diabetes Mellitus, Kidney Diseases	0.0150773169910305
3',4',5',5,7-Pentamethoxyflavone	145	Neoplasms, Hypertension, Diabetes Mellitus, Atherosclerosis, Kidney Diseases	0.0236906152690228
Epiberberine	138	Neoplasms, Hypertension, Diabetes Mellitus, Atherosclerosis, Myocardial ischemia	0.088972815749996
Syringetin	134	Neoplasms, Hypertension, Diabetes Mellitus, Atherosclerosis	0.0169184212986943
3,4-Divanillyltetrahydrofuran	122	Neoplasms, Hypertension, Diabetes Mellitus, Atherosclerosis	0.069015403805052
Anhydrosecoisolariciresinol	122	Neoplasms, Hypertension, Diabetes Mellitus, Atherosclerosis	0.073533086946177
Cyclo (L-Prolyl-L-tyrosine)	122	Neoplasms, Hypertension, Atherosclerosis, Diabetes Mellitus	0.073927521122302
Cyclo(Pro-Phe)	120	Neoplasms, Hypertension, Atherosclerosis, Diabetes Mellitus	0.0338796674550102
Urolithin B	119	Neoplasms, Hypertension, Diabetes Mellitus, Myocardial ischemia, Atherosclerosis	0.065172720058097
Auraptenol	114	Neoplasms, Myocardial ischemia, Kidney Diseases, Immune System Diseases, Hypertension, Diabetes Mellitus	0.00989760039264998
[(1R,2S)-1-(1,3-benzodioxol-5-yl) -2-methyl-3-oxobutyl] 4-hydroxy-3-methoxybenzoate*	111	Neoplasms, Hypertension, Diabetes Mellitus, Atherosclerosis	0.0240781024850327
Vitrofolal A	111	Neoplasms, Hypertension, Atherosclerosis, Diabetes Mellitus	0.089686576410831
Trijugin A	110	Neoplasms, Hypertension, Diabetes Mellitus, Atherosclerosis, Kidney Diseases, Myocardial ischemia	0.082417060442795

Name	The number of associated targets	Potential physiological activity	P-value
Phenylacetyl-L-glutamine	106	Neoplasms, Hypertension, Diabetes Mellitus, Atherosclerosis, Kidney Diseases, Myocardial ischemia	0.095895031458715
Coniferyl alcohol	101	Neoplasms, Diabetes Mellitus, Hypertension, Atherosclerosis	0.0520472250833688
Glycyl-tryptophan	101	Neoplasms, Hypertension, Atherosclerosis, Diabetes Mellitus	0.05106455435413
Cyclo(Pro-Val)	100	Neoplasms, Hypertension, Diabetes Mellitus, Atherosclerosis, Kidney Diseases	0.0357046261113931
Cyclo(D-Val-L-Pro)	99	Neoplasms, Hypertension, Diabetes Mellitus, Atherosclerosis, Kidney Diseases	0.0144730031996289
Phe-Ala	82	Neoplasms, Hypertension, Diabetes Mellitus, Atherosclerosis, Kidney Diseases	0.092041527661972
Cyclo(Pro-Pro)	75	Neoplasms, Hypertension, Diabetes Mellitus, Atherosclerosis	0.011500917720065
Emodin	71	Neoplasms, Hypertension, Diabetes Mellitus	0.00582099861315446
