# Supplementary material for: Wheat Bran Stir-Frying Reshapes the Metabolome of Euryale Ferox Seeds and Predicts Enhanced Bioactive Potential for Spleen and Kidney Tonifying
Source: Metabolites. 2026 Sep 10;16(9):669. doi: 10.3390/metabo16090669 (PMC13609870; doi:10.3390/metabo16090669)
Supplement: Supplementary file 1 [file metabolites-16-00669-s001.zip › Table S3.pdf]

Table S3. Re-docking RMSD results for all ten target proteins

Target	PDB ID	RMSD (Å)
AKR1B1	8FH8	0.6949
AKT1	8UW9	1.86807
ALOX5	6N2W	0.919315
GSK3B	9X2Q	1.6407
IGF1R	8PYN	1.21265
MMP2	7XJO	0.620311
MMP9	8K5Y	1.07625
PARP1	7ONS	0.740803
PIK3CG	6AUD	1.40999
PTGS2	5F19	0.9742
